# Turkish University seniors' knowledge of and opinions on fertility and expectations of having children

**DOI:** 10.4314/ahs.v18i1.22

**Published:** 2018-03

**Authors:** Nebahat Özerdoğan, Burcu Yilmaz

**Affiliations:** Department of Midwifery, Faculty of Health Sciences, Eskisehir Osmangazi University, Eskisehir, Turkey

**Keywords:** Fertility, knowledge, students, parenthood status

## Abstract

**Background:**

In recent years the trend of postponement of child bearing has been increasing.

**Aim:**

To assess knowledge on fertility and expectations of having children in seniors at a Turkish University.

**Methods:**

The cross-sectional study sample (n=485) comprised senior students of the University in the branches of medicine, social sciences, and life sciences. Data were collected via a self-administered questionnaire in 2015.

**Results:**

The mean age of the students was 23.03±1.93 and 98.1% were unmarried. 88.45% of these students wanted to have children in the future. A higher percentage of females planned parenthood in the future than males. Males desired more children than females. Most students wanted to have their first child at age 25–29. Males wanted to have their first and last child later than did females. In case of infertility, 74.4% of females and 54.2% of males stated that they can benefit from assisted reproduction techniques. Students overestimated the success rate of assisted reproduction techniques. The percentage of students who were aware of the age at which fertility begins to decline was low.

**Conclusion:**

The expectations of seniors at a Turkish university to have children in the future are high, with insufficient knowledge on fertility.

## Introduction

Maternal age at first birth is an important predictor of general fertility level as much as maternal and child health and quality of life.^1^ Today, more females tend to postpone pregnancy plans to advanced ages.^2^ In Turkey, the average age at first birth is 22.9 years and is increasing. The highest age-specific fertility rate is observed in the age group 25–29 in the last 10 years (the age group 20–24 before that).^3^

Couples in high-welfare societies postpone having family until their fertility declines. Factors affecting this decision are increased contraceptive use, improvements in assisted reproductive techniques (ART), delayed marriages, increased divorces, desire to focus on career, and economic factors.^4^,^5^ Moreover, the expectation that children may be raised under better conditions at more mature ages may also be behind this trend.^2^

Postponing the age at first childbearing has an important impact on fertility and pregnancy. Pregnancy and childbearing at advanced age may lead to fetal and maternal complications, increases in morbidity and mortality rates, and diminished fertility and productivity rates.^6^ Fertility rates start to decline at age 30 in females and at age 40 in males. While the monthly probability of a woman under the age of 30 becoming pregnant is 20%, after the age of 40, this rate drops up to 5%.^7^,^8^ Endocrinal changes appearing with advanced age cause a diminished ovarian volume, reduction in the number of ovarian follicles, and decreased quality of oocytes. Furthermore, there may be an increase in chromosomal changes and related fetal abnormalities in infants born to older mothers.^9^ As in females, the tendency among males too is to postpone parenthood until later age. Males over the age of 35 display a distinct decline in semen parameters and after the age of 50, there is a noticeable reduction in fertility.^8^

Childbearing may be postponed to later age in individuals attending university programs due to various reasons, mainly due to career plans. This may cause fertility problems associated with decreased reproductive capacity and involuntary childlessness.^4^ The studies conducted on University students show that the majority of the students plan to have children in the future, but they lack sufficient knowledge on fertility.^4^,^10^,^11^,^12^ The present study aimed to evaluate the knowledge and views of University seniors on fertility as well as their expectations regarding their parenthood plans for the future.

## Method

This cross-sectional study was conducted on senior University students between May and September, 2015. The study comprised senior students who received undergraduate education at Eskisehir Osmangazi University in Turkey.

### Study sample

The sample size included 256 students with a statistical power of 90%, and confidence interval of 0.95 using the data of Lampic et al. with the PASS 11 program.^4^ One program was selected by lot from each faculty in the branches of medicine, social sciences, and life sciences( seven faculties in total). All of the final year students attending these programs were included in the study (n=602). Of these students, 57 declined participation in the study and 60 provided invalid responses in the questionnaire. Therefore, the study was conducted on a total of 485 students (262 females and 223 males). The students were divided into three main groups based on their departments: medical studies (n=73), social sciences (n=267), and sciences (n=145).

### Data collection

Data was collected using a self-administered questionnaire prepared according to the literature.^4^,^6^,^10^ It was composed of three parts (20 questions). The first part included five questions to determine students' socio-demographic characteristics; the second part included seven questions to determine students' expectations of having children; and the third part included eight questions (six open-ended) to determine the extent of students' fertility-related knowledge. Students were surveyed in the classroom.

### Ethical considerations

The study was approved by the Institutional Review Board of the researchers' institution (IRB; EOU-15-05-28/10). Students were informed of the study and verbal consent was obtained.

### Statistical analysis

The analyses were performed using SPSS 21.0 program. Data analysis was performed using descriptive statistics, ANOVA, chi-squared, and independent samples t-tests. A p value of <0.05 was considered statistically significant.

## Results

The mean age of the students was 23.03 (SD = 1.93, range 19–37). Of the students, 15.1% were from medical branches (n=73), 55.1% from social sciences (n=267), and 29.9% from life sciences (n=145) (Table 1). All males and 97.3% of females did not have children, and 1.9% of them were married.

**Table 1:** Ages and branches of students by gender (n=485)

Characteristic	Women (n:262) n (%)	Men (n:223) n (%)	Total n (%)
**Age**			
≤22	138 (52.7)	69 (30.9)	207 (42.7)
>*22 years*	124 (47.3)	154 (69.1)	278 (57.3)
± *SD*^a^ *(Min.-max.)*	22.69 ±2.01 (19–37)	23.42±1.77 (20–34)	23.03±1.93 (19–37)
**Type of curriculum**			
*Medical studies*	46 (17.6)	27 (12.1)	73 (15.1)
*Social studies*	167 (63.7)	100 (44.8)	267 (55.1)
*Sciences*	49 (18.7)	96 (43)	145 (29.9)
**Total**	**262 (100)**	**223 (100)**	**485 (100)**

a Standart Deviation (SD)

### Students' plans for parenthood

88.45% of the students wanted to have children in the future. The number of males who did not want to have children was higher than the number of females who did not want to have children (p=0.001). The average number of children that females planned to have (2.33) was lower than that of males (2.53) (p=0.020). It was found that 72.5% of females and 48.6% of males planned to have their first child in the age range of 25–29. The percentage of males (34.7%) who wanted to have their first child between the ages of 30–34 was higher that of females (p<0.001). The age of having the last child was 30–34 in 50.2% of females and 35–39 in 37.8% of males. This rate was lower in females (p<0.001). In case of infertility, 74.4% of females and 54.2% of males stated that they can benefit from ART. The percentage of males who would give up the idea of adopting or having a child was higher (Table 2) (p<0.001).

**Table 2:** Students' intentions about having children, by gender

Students' intentions about	Women	Men	p
having children	n (%)	n (%)	
**Want to have children in the future**			
**(Totally: 88.45%)**			
***Yes***	239 (91.2)	190 (85.2)	**0.001**^a^
***No***	1 (0.4)	13 (5.8)	
***Don't know***	22 (8.4)	20 (9.0)	
**Desired number of children**			
**(Mean^b^: 2.42±0.892; n=437)**			
**1**	26 (9.9)	8 (3.6)	**0.020**^c^
**2**	138 (52.7)	118 (53.4)	
**3**	51 (19.5)	40 (18.1)	
**4**	18(6.9 )	23 (10.4)	
**5**	6 (2.3)	9(4.1)	
**Don't know**	23 (8.8)	23 (10.4)	
**Mean± *SD***	2.33 ± 0.862	2.53 ±0.916	
**Desired age at first child (years)**			
***<20***	1 (0.4)	2 (0.9)	**0.001**^a^
***20–24***	25 (9.5)	12 (7.6)	
***25–29***	190 (72.5)	108 (48.6)	
***30–34***	33 (12.6)	77 (34.7)	
***35–39***	0 ( 0)	6 (2.7)	
***≥40***	0 ( 0)	1 (0.5)	
***Don't know***	13 (5)	16 (7.2)	
**Desired age at last child (years)**			
***20–24***	1 (0.4)	0 ( 0)	**<0.001**^a^
***25–29***	19 (7.3)	11 (5.0)	
***30–34***	131 (50.2)	66 (29.7)	
***35–39***	57 (21.8)	84 (37.8)	
***40–44***	6 (2.3)	30 (13.5)	
**≥45**	1(0.4)	9 (4.1)	
***Don't know***	46 (17.6 )	22 (9.9)	
**Behavior in case of infertility**			
***Undergo IVF***^d^	189 (74.4)	117 (54.2)	**0.001**^a^
***Adoption***	33 (13.0)	49 (22.7)	
***Choose not to have children***	22 (8.7)	35 (16.2)	
***Don't know***	10 (3.9)	15 (6.9)	

a Chi-square test was performed

b The average of all students

c Independent samples t-test was performed.

d In vitro fertilization (IVF)

### Students' knowledge of fertility

The percentage of medical students who knew the most fertile age of females and the answers to questions related to the rate of infertile couples in Turkey was higher than that of the other students (p<0.001, p=0.022). The percentage of students who correctly answered questions on the age at which female fertility declines was low. The percentage of students who correctly answered question on pregnancy rate due to unprotected sexual intercourse between individuals younger than age 25 was also low. There were no differences among the student of different departments (p>0.05). The percentage of students who correctly answered the question on the age at which female fertility declines was 17.5%. The percentage of male students who correctly predicted the chance of pregnancy with ART was higher (p=0.025) (Table 3).

**Table 3:** Questions and answers regarding information about fertility, broken down by gender and department

Questions and answers†	Department (%)					Gender (%)			

	Medical	Social	Sciences	Total	p‡	Women	Man	Total	p‡
	n=73	n=267	n=145	n=485		n=262	n=223	n=485	
At what age range are women the most fertile? (20–24 **ages**)									
***Correct***	72.6	53.9	44.8	54.0	**.001**	56.9	50.7	54.0	>.05
***False***	27.4	46.1	55.2	46.0		43.1	49.3	46.0	
***Mean***	25.0	25.6	26.5	25.8		25.4	26.42	25.80	
At what age is there a slight decrease in female fertility? (25– 29 **ages**)									
***Correct***	0.0	0.4	0.7	0,4	>.05	0.4	0.4	0.4	>.05
***False***	100	99.6	99.3	99,6		99.6	99.6	99.6	
***Mean***	36.8	39.1	37.4	38.3		38.2	38.4	38.3	
At what age is there a marked decrease of female fertility? (35– 39 **ages**)									
***Correct***	15.1	18.4	17.2	17,5	>.05	18.7	16.1	17.5	>.05
***False***	84.9	81.6	82.8	82.5		81.3	83.9	82.5	
***Mean***	41.8	42.1	40.5	41.6		41.8	41.4	41.6	
What is the chance/risk (%) of a pregnancy occurring when a young man and woman under 25 years of age have **unprotected** **intercourse?** (30– 39 **%**)									
***Correct***	4.1	3.4	4.8	3.9	>.05	3.8	4.0	3.9	>0.05
***False***	95.9	96.6	95.2	96.1		96.2	96.0	96.1	
***Mean***	67.0	70.4	60.4	66.9		70.1	63.4	66.9	
How many couples in Turkey are involuntarily childless? Rate of **infertile couples in** **Turkey (**10–20 **%**)									
***Correct***	53.4	36.0	42.8	40.6	**.022**	40.5	40.8	40.6	>0
***False***	46.6	64.0	50.2	59.4		59.5	59.2	59.4	.05
***Mean***	12.9	21.1	17.7	18.7		22.8	14.3	18.7	
Success rate with ART (20–29 %)									
***Correct***	9.6	8.2	9.7	8.9	>.05	6.1	12.1	8.9	**0.025**
***False***	90.4	91.8	90.3	91.1		93.9	87.9	91.1	
***Mean***	47.7	45.4	44.3	45.4		47.8	43.1	45.4	

Note.ART= Assisted reproductive technologies ;

† The ‘correct’ answersare in brackets;

‡ Chi-square test was performed

## Discussion

The study evaluated fertility knowledge and expectations of having children of final year University students, and the majority of the students expressed their desire to have children (88.45%). The percentage of students planning parenthood in the future was 96%–97% in two studies^4^,^10^ conducted in Sweden and 94% in Finland.^11^ The percentage of students planning parenthood in the future was 90% in the USA,^12^ which is close to the results of the present study, and this percentage was lower among students in Ukraine,^13^ China,^5^ and Austria (80%–77%).^6^ The different percentages of students planning parenthood in the future in different countries can be explained by socio-economic conditions and cultural characteristics. Studies conducted in Ukraine have attributed the low rates of students planning parenthood in the future to economic instability and current policies.^13^ In the present study, the percentage of students planning parenthood in the future differed with gender, which is different from[PJT1] the studies by Lampic et al.^4^ and Nouri et al.6, but in parallel with the studies by Ekelin et al.^10^ and Mogilevkina et al.^13^ The number of female students planning parenthood was higher than the number of males planning parenthood. Fertility raises the social status of females in communities with patriarchal structure. With respect to gender roles, motherhood increases approval and acceptance of a woman within the community.^14^ This may be a factor in increasing the fertility desire of females.

In the study, the average number of children desired by university students was 2.42. This number is similar in different countries (2–3 children).^4^,^10^,^11^ The study conducted with medical students in Ukraine showed that the average number of children desired by females (average 1.6 children) and males (average 1.4 children)was lower.^13^ The total fertility rate in Turkey (2.14) has been rapidly declining since 1970s.^3^,^15^ The Turkish government suggests that families should have at least three children in terms of protecting population size. The number of children that students want to have is between the recommended value and the current total fertility rate. In our study, the average number of children that females planned to have was less than that planned by males. This is because females may have concern that their career and work life will be negatively affected by pregnancy and child care associated with their motherhood role. It is because of the fact that a mother is the most responsible person for the child care in the Turkish society. Fathers usually do not take responsibility for childcare.^16^ The number of children that Austrian students want does not vary according to gender. 6 However, the rate of female students who want to have three or more children in Sweden, Italy, and Finland is higher.^4^,^10^,^17^

In the present study, males postponed the age of having the first and the last child to advanced ages compared with females. The age range at which students mostly want to have their first children is age 25–29. However, the rate of those who wanted to have their first child between the ages of 30–34 was approximately three times higher in males than in females. The average maternal age at first birth in Turkey (age 22,^9^) is lower than our findings. However, as the education levels of females have increased, the average age at first birth has also increased.^3^ This observation supports our findings. The findings on the age at which the first child was desired was similar between our findings and those of other studies. Furthermore, our finding that that male students delay having their first child to advanced ages than do female students is also in line with the results of other studies.^4^,^10^,^11^,^17^ According to their gender roles, males consider themselves primarily responsible for the livelihood of the house.^16^ This may explain the fact that males want to have children after a period when they have regular and sufficient income.

In case of possible infertility, students mostly preferred to have children with the help of ART. Then, they preferred to adopt a child. At last, they preferred to give up having a child. These findings are in line with results of other studies.^4^,^10^,^11^ In such a case, female students stated that they preferred to benefit from ART. On the other hand, male students stated that they preferred to adopt a child or give up having a child. The reason why ART is more preferred in females is believed to be the biological desire to have their own child and fulfill the role of motherhood. In other studies, the rate of giving up having a child in the case of infertility is higher in males than in females.^4^,^10^,^12^ Lampic et al.^4^ and Peterson et al.^12^ have shown that the rate of those who consider adopting a child is higher in females than in males. This is considered to be related to the culture of the society.

In this study, except for the two questions on the age at which females are the most fertile and rate of infertile couples in the country, the correct answer rate of the students to other questions was very low (0.4%–17.5%). These two questions were answered correctly by more medical students. Similar to the study conducted by Lampic et al.^4^, half of the students knew the age at which females are the most fertile and the rates did not vary with gender. When compared with other studies,^1^,^4^,^6^,^12^ the percentage of knowing the age at which fertility begins to decline and markedly declines was lower and this rate did not differ according to the department of students or their gender. Similar to other studies,^1^,^4^,^6^,^12^ students estimated the age at which fertility begins (average 38.3 years) to decline and markedly declines (average 41.6 years) at a higher value than it should be. The values estimated by students on the rate of pregnancy that can occur as a result of unprotected sexual intercourse between a man and a woman under the age of 25 were higher than they should be. These findings are in line with results of other studies.^1^,^4^,^12^. These results show that students are not sufficiently aware of the decline in fertility with age. Furthermore, students evaluate the chance of pregnancy to be higher than it is in reality. The rate of those who correctly predicted the success rate of ART was higher in males. Similar to the other studies,^4^,^12^ students expected higher achievement from ART than it should be.

## Conclusion

Students' knowledge on fertility is inadequate. The percentage of students who were aware of the age at which fertility begins to decline and markedly declines was low. The students evaluate the chance of pregnancy to be higher than it is in reality. They have excessive expectations for pregnancy to occur and of the success rate of ARTs. A significant proportion of students, particularly males, plan to have their first child when fertility begins to decline. This may cause problems in students' fertility and child bearing plans. It is thus important to make reproductive health services to which students can consult about fertility widely accessible and ensure that nurses and midwives are actively involved in these services.
